# A Word of Caution against the Stigma Trend in Neglected Tropical Disease Research and Control

**DOI:** 10.1371/journal.pntd.0000445

**Published:** 2009-10-27

**Authors:** Joan Muela Ribera, Koen Peeters Grietens, Elizabeth Toomer, Susanna Hausmann-Muela

**Affiliations:** PASS International vzw., Tessenderlo, Belgium; Ghana Health Services, Ghana

**Learning Points**

1. Stigma kills. It kills socially because it excludes affected persons from social life by reducing social networks and possibilities to find work, marriage partners, etc.
2. Stigma also kills literally, as a result of stigma-related social exclusion and treatment delay. Stigma may hinder treatment-seeking in two different ways: (1) through fear or shame to be seen in public places, e.g., health centers; and (2) as a result of social exclusion, through reducing economic capital necessary to cope with illness costs.
3. Stigma is a powerful element in determining health behavior and is one reason for social isolation and exclusion. But be careful: it is not the only one. A narrow focus on stigma bears the danger of diverting attention away from structural, economic, and political factors affecting health behavior. Apart from social inequalities, other factors like direct and indirect costs of the disease and its treatment can lead to social isolation.
4. Whether or not it is stigma that limits health-seeking behavior or leads to a delay in treatment or to social isolation, this has important implications for health interventions. If stigma *is* relevant, sensitization campaigns are justified. If stigma is *not* or of little relevance, other interventions should be prioritized.
5. Social science research in neglected tropical diseases does not need new fashions, but solid conceptual frameworks of health-seeking behavior or vulnerability that study all relevant factors.

A man, suffering from an advanced stage of Buruli ulcer disease, is sitting on a wobbly stool repairing an old fishnet. As he limps to add the rags of old clothes he will use later as bandages to a pot of boiling water, the stained bandage covering his oozing wound on his leg and foot becomes apparent. *“What happened? I was always good in school and worked hard on my fields. I was successful. But it's the jealousy! Someone in the village cursed me with atom (Buruli ulcer) and now I'm here like this! Since then, four years ago, I've had to give up my studies, my wife has left me. It was too much, too long. And now I have to pay for the schooling of my two children as well as for my treatment. I can not participate in the work groups anymore because of my condition, and so I have no one to help me with my fields which are ruined because of the time I spent at the hospital, waiting for healing… I do have a large family but I have to take care of myself alone now, without their help. But, my life isn't over! I'm still young, I'm strong. My life isn't over!”* He is alone. Waiting.

## Introduction

The introductory account is taken from our field notes of an interview with a Buruli ulcer disease (BUD) sufferer in Central Cameroon in February of 2006 who, after seeking numerous treatments, including specialized BUD biomedical treatment, finds himself alone, abandoned by family and friends, and with few prospects. Was it the stigma associated with a disease that is locally attributed to witchcraft that caused this man's abandonment, or were there other elements involved that account for his present situation? At first glance, it might indeed seem that in this case, stigma struck again. But did it?

The discussion on stigma is gaining ground in the literature about neglected tropical diseases [1],[2]. Two neglected diseases, leprosy and syphilis, are among the first described as “repulsive” [3], and both can be considered prototypes of stigmatizing diseases. Aside from leprosy [4]–[8] and sexually transmitted diseases [9], stigma has been associated with hematuria in urinary schistosomiasis [10], lesions of onchocercal skin disease [11]–[14], scabies [15],[16], leishmaniasis [17], and Buruli ulcer [18],[19].

Such labeling of some neglected tropical diseases as “stigmatizing” may indeed increase political commitment to these diseases [2], but it also bears some risks. Research can easily end up confirming previous assumptions, i.e., that it is the social stigma associated with the disease that leads to social isolation, hampers access to care, and reduces treatment adherence. Although this may be the case, in contexts with high levels of poverty and poor health services, other factors, such as high treatment costs, tedious travel to health centers, and long hospital admittances, must not be ignored. In fact, the task of social science research is to scrutinize all possible explanations, without being blinded by the apparently obvious. Moreover, a narrow focus on stigma is not only a methodological pitfall, but it is also a dangerous way of taking responsibility for poor health care attendance away from the political and economic domains and placing the blame on “culture”.

The aim of this paper is to caution against an all too euphoric use of “stigma” in neglected tropical diseases research. In order to prevent losing sight of the variety of possible explanatory options, we encourage the use of a “falsificationist” approach with systematic hypothesis testing that incorporates, but is not restricted to, stigma.

## Stigma Kills

As Van Brakel [7] argues in his literature review on leprosy and stigma, with conditions like leprosy, AIDS, epilepsy, schizophrenia, etc., the stigma may be worse than the disease itself. Stigma can produce an often irrational rejection of its victims by the stigmatizers, and also by the stigmatized themselves (self-stigma) and their allies. For this reason stigmas are often labeled as “social killers” since this rejection can lead to loss of social networks, loss of work, difficulty in finding marriage partners, divorce, loss of reputation, discrimination, isolation, ostracism, etc. [20]–[23].

Furthermore, stigma produces and reproduces social inequalities and structures of exclusion. According to Sen [24], “not to be able to mix with others might directly impoverish a person's life, and also, additionally reduce economic opportunities that come from social contact. Indeed, quite often different aspects of capability deprivation and social exclusion may go together” (p. 14). Precisely because stigma negatively affects the economic and social capital of households—which are key for dealing with illness—one could assume that stigma limits access to health care both by increasing feelings of fear and shame as well as by reducing people's capabilities to successfully obtain appropriate treatment.

Understandably, victims of stigmatizing diseases may opt not to attend health centers since their presence may reveal their condition, with the consequent exacerbation of disease and suffering that result from treatment delay [1]. Various studies show that stigma associated with sexually transmitted diseases directly or indirectly hinders access to public health clinics, and hence stigma is an important disincentive to seeking treatment [25],[26]. Similar arguments have been used in HIV/AIDS research [27], epilepsy [28], and leprosy [4].

Health centers, above all those that specialize in sexually transmitted diseases or leprosy, are public spaces, where the simple act of attendance gives evidence or discloses the stigmatizing condition of the user. This may explain why many people prefer attending health centers or hospitals far away from their residences in order to remain anonymous—a coping strategy that has been described by Pearson [29] and Barrett [8] for leprosy cases. Therefore, treatment delay can be the perverse consequence of one of the main strategies for coping with stigma: the occultation and masking of stigma [30].

## Treatment Delay beyond Stigma

However, that stigma hampers access to health care poses in fact a paradox. Why should one avoid efficacious treatment if the disease causes not only physical suffering but also discrimination and additional pain due to its stigma? Why should stigma be an obstacle rather than an incentive for initiating or complying with treatment?

As we have seen in the previous section, stigma, and fear of diagnosis, can indeed pose a barrier, particularly when consequences are potentially devastating, such as loss of work, shame, divorce, or abandonment. However, one should not uncritically accept the inhibiting role of stigma in accessing health and thereby neglect other relevant factors, such as the lack of efficacious drugs or the lack of resources for coping with illness costs.

For example, arguing against the increasing use of stigma as an all-encompassing explanation in HIV/AIDS behavioral research, Castro and Farmer [31] pointed out the following: “Where is the evidence that stigma is a barrier for access to treatment, when in 2002 less than 5% of persons affected by AIDS in poor countries had access to highly efficacious antiretroviral therapy?” (p. 53). They reiterate: “What is the motivation for learning one's serostatus when there is no possibility of being treated for opportunistic infections?” (p. 56). If efficacious treatment was available, would they not use it if they could afford it?

Castro and Farmer furthermore suggest that it is not stigma, but the limited accessibility of efficacious treatment and cost barriers that are the main factors explaining people's attitudes towards diagnostic tests, abandonment of therapies, or non-treatment. They also show that the implementation of effective therapy for mothers in the Dominican Republic has helped diminish patients' stigmatization. An AIDS program in rural Haiti also reported a sharp decline in AIDS-related stigma [31].

Likewise, Pearson [29], in her study on leprosy in Nepal, concluded that the poor quality of care at the established leprosy health centers, rather than the fear of being locally known as having leprosy, is decisive for women not to attend the new services. Similar findings have been reported for cancer where new treatments and the increasing likelihood of patients' survival have been demystifying the illness [3].

## Social Isolation beyond Stigma

One risk of inadequately using the concept of “stigma” in public health consists of attributing social isolation or exclusion to the stigmatizing character of the illness, without considering other possibilities. BUD is a neglected tropical disease that is considered highly stigmatizing [18],[19] due to its visible lesions but also due to the local attribution of BUD to social transgressions and witchcraft [19]. In a study we carried out in Cameroon [32], BUD exhibits all the signs of being a stigmatizing disease: BUD is clearly associated with transgressions of social norms, such as theft and witchcraft, and many of its sufferers are abandoned at the hospitals, leading to the patients' consequent abandonment of free-of-charge hospital treatment. Undoubtedly, some of the physical characteristics of BUD (such as the bad smell of the ulcer or the deformations of the effected limbs) are unsightly and have the potential of inspiring feelings of insecurity, shame, and discomfort to the patient. And similar to the account above of the man with BUD who had been financially, socially, and emotionally drained due to his illness, many people with BUD find themselves alone, without prospects, and waiting for healing. Nonetheless, a detailed analysis of the BUD patients admitted in the Ayos and Akonolinga Hospitals in Central Cameroon revealed that isolation had other roots: the social isolation patients faced was due to a household coping strategy attempting to avoid plunging the household into a spiral of impoverishment [32].

Although at the Ayos and Akonolinga hospitals treatment is free of charge, the cost burden of hospitalization for BUD accounts for 25.2% of households' yearly income [32], more than double the 10% commonly considered catastrophic for the household economy [33]. Treatment costs consist of productivity time lost for patients and caretakers, transportation expenses and feeding costs, and to a lesser extent the purchase of soap, bandages, and extra medicine. Distance of the hospitals from the communities and the long periods of time that patients must remain at the hospital—median treatment time for all patients treated at both hospitals during the period 2002–2007 was 157 days [32]—are the underlying causes for such a high cost burden of BUD in Central Cameroon. Additionally, absence in the community generates an extra economic burden, as it hinders continued participation in community social networks such as work groups and savings clubs.

Understandably, 62.6% of households ceased providing financial support for patients and making regular visits to the hospital. In fact, for patients who were not isolated, the cost for their households during the healing process (a median of €105.9) was 8.6 times higher than for isolated patients (a median of €12.4) [32]. Moreover, patients mentioned social isolation as the principal reason for abandonment of biomedical treatment [32].

With regard to delay, the difficulty in initially distinguishing signs and symptoms of BUD from everyday insect bites or abscesses, the lack of familiarity with and accurate diagnosis of BUD at non-specialized hospitals, and the overall difficulty in successfully treating BUD play significant roles in late stage arrival at specialized BUD units. However, delay is mainly related to household attempts to minimize or avoid the debilitating costs associated with treatment.

## Implications for Public Health

In a nutshell, stigma should not be an uncritical explanation for treatment delay or abandonment, but a hypothesis that has to be carefully tested in the field. To do so correctly is pivotal for designing adequate public health interventions.

In those cases where the obstacle for seeking adequate preventive or curative care *is* indeed stigma, public health programs do well in fighting stigma through sensitization campaigns. The classical approach is the Information-Education-Communication (IEC) campaigns that include culturally adapted messages about illness and its treatment. In the words of Stienstra [19] in her recommendation for improving BUD detection and control, “educational programs should be developed, not only because they could help in the detection of cases in an earlier stage of the disease, but because they might also lower stigma”. Such campaigns aim at changing attitudes, both of the society towards the affected and of the stigmatized themselves.

However, in those cases where stigma proves *not* to be a major obstacle, other strategies are required. Debacker et al. [18], in their study on BUD in Benin, proposed the creation of “regional centers that allow patients easy access to treatment with short travel distances and low treatment costs, coupled with educational sessions. This proximity would render the follow-up of patients easier and be a source of new information on the disease for the population”. Similarly, in our study on BUD in Cameroon, where social exclusion is above all a result of households' coping strategies to avoid falling into the “medical poverty trap” [34], the health policy that is most likely to be successful is to improve treatment access, e.g., through a strategy of decentralization of treatment [32].

Besides sensitization campaigns, improving the quality of care and access to effective biomedical resources should be a major focus. Castro and Farmer [31] suggest that good access to treatment helps foster an environment which, step by step, is likely to counteract the vicious circle of illness, stigma, and poverty.

In order to improve our understanding of stigma and its psychological and socioeconomic impact on access to care, it is of paramount importance to situate stigma in relation to other factors and to contextualize it in broader conceptual frameworks, be it in health-seeking behavior, vulnerability, structural violence, or social exclusion.

## Conclusions

Since the 1990s, “beliefs” have dominated the behavior change literature of international health projects. Reflecting back, the “beliefs” boom was rooted in an exaggerated enthusiasm of identifying “wrong beliefs” as the barrier to access to health care. Consequently, well-designed IEC messages were regarded as the key to correcting people's behavior. The overemphasis of beliefs, situating the access problem at the level of “cultural obstacles”, entirely disregarded a person's socioeconomic status and capacity to cope with health care costs, or structural factors like health care infrastructure, quality of care, etc. Fortunately, today, we have moved to a more integrated view, where cultural factors are analyzed together with social, economic, political, and environmental factors.

Spilling over from HIV/AIDS and tuberculosis work, stigma bears the danger of becoming a new “cultural boom” in neglected tropical diseases if not systematically analyzed. Caution is required against overemphasizing stigma as the sole factor responsible for limited health care access. As we have shown, various other factors can lead to reduced health care and social isolation. Furthermore, improved availability and especially efficacy of health care resources does not only favor access to health care, but can also play a role in reducing stigma [31].

Uncritical attribution of social exclusion and lack of access to care to stigma might detract from other fundamental causes. The example of BUD in Cameroon [32] has clearly shown that social isolation and treatment abandonment is strongly linked to economic constraints of caretakers and families, rather than to stigma. Therefore, well-designed awareness campaigns with the aim of reducing stigma are unlikely to lead to successful behavior change if they are not accompanied by the improvement of people's capacities to cope with the economic costs of illness.

Again, the “stigma pitfall” shows interesting parallels to the “beliefs pitfall”. Looking only at beliefs in order to explain patients' visits to traditional healers can easily detract from other, substantially more important, reasons for not attending health centers, e.g., possibly unaffordable, poor quality, or unavailable biomedical treatment. Hence, looking at access to health care through an isolated “stigma” lens can decontextualize the problem and lead to ineffective solutions.

Stigma is a powerful element in determining the course of a disease and its social burden [1],[2], and merits attention. Nevertheless, while supporting current appeals to place stigma on researchers' and implementers' agendas, we strongly caution against uncritically using “stigma” as an all-explaining concept in public health.

With this appeal for caution, we hope to contribute to stimulating the discussion on stigma and to encourage careful analyses including all factors that play a role in hindering access to health care.

## Supporting Information

Alternative Language Abstract S1 Translation of the abstract into Spanish by Joan Muela Ribera
(0.03 MB DOC)
