# Supplementary material for: A Word of Caution against the Stigma Trend in Neglected Tropical Disease Research and Control
Source: PLoS Negl Trop Dis. 2009 Oct 27;3(10):e445. doi: 10.1371/journal.pntd.0000445 (PMC2761539; doi:10.1371/journal.pntd.0000445)
Supplement: Alternative Language Abstract S1 — Translation of the abstract into Spanish by Joan Muela Ribera (0.03 MB DOC) [file pntd.0000445.s001.doc]

**Resumen**

**Una llamada de atención contra la moda del estigma en la investigación y control de las enfermedades tropicales olvidadas**

**Joan Muela Ribera**

El concepto de ‘estigma’ está ganando, merecidamente, peso en el ámbito de la investigación de las enfermedades tropicales olvidadas. Su presencia supone un avance no solo importante, sino también revelador, en cuánto que muestra cómo los factores sociales afectan el acceso a la salud. Pero atención, porque este enfoque en el papel del estigma como obstáculo a la salud y fuente adicional de sufrimientos para las personas enfermas corre el riesgo de convertirse en una nueva moda, oscureciendo otros factores sociales realmente relevantes para comprender la problemática del acceso al tratamiento. Basándonos en la literatura sobre estigma y en nuestro propio trabajo de campo sobre la exclusión social de pacientes con úlcera de Buruli en Camerún, en este artículo cuestionamos la ‘moda’ que sistemática y acríticamente tiende a atribuir el aislamiento social y las limitaciones en el acceso a la salud al estigma de la enfermedad. El estigma debe ser un factor a tener en cuenta, pero los investigadores deben tener en cuenta todos los elementos potencialmente relevantes para el acceso a la salud, evaluándolos y sospesándolos de manera escrupulosa. Es decir, abogamos por una perspectiva falsacionista, que impida el error metodológico de enfocar la investigación con las ‘lentes’ exclusivas del estigma.
